# 236 Outbreak of Sarocladium kiliense endophthalmitis after cataract surgery associated with contaminated off-label trypan blue use in Japan

**DOI:** 10.1017/ash.2026.10612

**Published:** 2026-06-23

**Authors:** Jaclyn Bernadzikowski, Rachel Weber, Maureen Bolon, Sandra Reiner, Laura Bardowski, Grace Barajas, Jacqueline Maniscalco

**Affiliations:** 1 Northwestern Memorial Hospital

## Abstract

**Background** Illinois has one of the highest regional Candida auris burdens nationwide. Our hospital has a surveillance protocol for Candida auris that dictates all patients with an admission in the prior 6-months to a skilled nursing facility (SNF), long-term acute care hospital (LTACH), and/or inpatient rehab (IR) be tested on hospital admission. Additionally, three high-risk units at our institution have an increased surveillance protocol that includes testing all patients on admission, transfer, and weekly for C. auris. Two years of first-positive C. auris cases were reviewed to investigate if our surveillance protocol was capturing all patients with C. auris present on admission. Method A retrospective chart review was conducted using our electronic database and medical record (EMR). All admitted inpatients between January 1, 2023 to December 31, 2025 with a first-positive Candida auris laboratory specimen were included. Prior positive laboratory tests and outside facility encounters or transfers were manually reviewed from the EMR. Results A two-year data review identified 121 unique patients with positive Candida auris clinical or surveillance results. Of the 121 patients, 99 (81.8%) had no documented history (NDH) or prior positive laboratory results for C. auris. These cases were categorized as hospital onset (HO) 74/99 (74.7%) or present on admission (POA) 25/99 (25.3%). Of the 25 POA/NDH patients, 14 (56%) were directly admitted from a SNF, LTACH, or IR. Of the 25 POA/NDH patients, 17 (68%) had exposure to SNF, LTACH, and/or IR in the 6 months prior to admission. There were 8/25 (32%) patients admitted directly from home or outside hospital (OSH) and had no SNF, LTACH, or IR admissions 6 months prior. These 8 patients did not qualify for surveillance testing when screened for admission. Of the 8 patients, 6 (75%) had an admission to an OSH in the 6 months before surveillance screening. Conclusion The 8 POA/NDH cases without exposure to SNF, LTACH, or IR raise the question of whether including OSH encounters into our screening questionnaire could increase the accuracy of our C. auris surveillance protocol. We have demonstrated that exposure to SNF, LTACH, and IR is a risk factor. Other regional hospitals should be encouraged to evaluate or begin surveillance protocols to help define whether hospital admissions are a risk factor for C. auris acquisition. Validating the accuracy of risk factors will help us better understand the epidemiology of C. auris and potentially prevent HO transmissions from missed POA cases.

